# Microbial Risk Assessment of Tidal−Induced Urban Flooding in Can Tho City (Mekong Delta, Vietnam)

**DOI:** 10.3390/ijerph14121485

**Published:** 2017-11-30

**Authors:** Hong Quan Nguyen, Thi Thao Nguyen Huynh, Assela Pathirana, Peter Van der Steen

**Affiliations:** 1 Center of Water Management and Climate Change (WACC), Vietnam National University—Ho Chi Minh City (VNU—HCM), Ho Chi Minh City 12345, Vietnam; n.huynh@un-ihe.org; 2 IHE Delft Institute for Water Education, 2611 AX Delft, The Netherlands; a.pathirana@un-ihe.org (A.P.); p.vandersteen@un-ihe.org (P.V.d.S.)

**Keywords:** health risk assessment, quantitative microbial risk assessment, urban flooding, water pollution

## Abstract

Public health risks from urban flooding are a global concern. Contaminated floodwater may expose residents living in cities as they are in direct contact with the water. However, the recent literature does not provide much information about this issue, especially for developing countries. In this paper, the health risk due to a flood event occurred in Can Tho City (Mekong Delta, Vietnam) on 7 October 2013 was investigated. The Quantitative Microbial Risk Assessment method was used in this study. The data showed that the pathogen concentrations were highly variable during the flood event and exceeded water standards for surface water. Per 10,000 people in contact with the floodwater, we found *Salmonella* caused the highest number of infections to adults and children (137 and 374, respectively), while *E. coli* caused 4 and 12 cases, per single event, respectively. The results show that further investigations on health risk related to flood issues in Can Tho City are required, especially because of climate change and urbanization. In addition, activities to raise awareness- about floods, e.g., “living with floods”, in the Mekong Delta should also consider health risk issues.

## 1. Introduction

Cities in developing countries, faced with rapid urbanization, encounter a number of problems that are connected to their development process. Among these, urban flooding and water quality pollution are among the major ones [1]. Increased imperviousness due to rapid urban densification, under-developed sewer systems, upstream flooding and tidal effects (in delta cities) are regular causes of urban flooding. Surface water pollution in urban areas comes from both point and diffuse sources [2]. Non or partially treated wastewater from domestic (and industrial) activities are clear pollution sources (i.e., point sources) discharging directly into receiving water bodies e.g., rivers and canals. A source of diffuse pollution is contaminated urban runoff, which increases along with the urbanization progress in developing countries [3].

Polluted water in the rivers, drainage canals and sewer systems can be mixed with floodwater on the streets, pavements, etc. during flood episodes and thus likely impact human health [4,5,6]. Infectious diseases are common issues for urban poor populations after flood events [1]. For example, people are exposed to microbial contaminants while walking through flooded roads, or playing with flooded water (kids) [7]. Different diseases are normally found related to flood events such as fecal–oral diseases, vector-borne diseases, rodent-borne diseases, acute asthma and skin rashes [8,9,10]. 

The risk of microbial contaminant has been investigated in Vietnam. For example, high microbial concentrations were observed in fertilizer and wastewater reused in agriculture [11]. In another example, total coliforms, *E. coli* and *Salmonella* spp. in wastewater for irrigation were 110-fold above Vietnamese agriculture standards and 260-fold above the World Health Organization (WHO)’s tolerable safety limits for unrestricted agriculture [12]. These pathogens have been considered potential diarrhea risk to farmers and consumers [13,14]. While a number of previous studies have highlighted human health risk in exposure to wastewater in agriculture, only limited studies have been made on the exposure to floodwater in urban areas.

Can Tho is located at the heart of the Mekong Delta and 75 km from the East Sea. It has a low and flat topography along the Hau River—a downstream branch of the Mekong River. Can Tho is the fourth most populous city in Vietnam, with a surface area of around 1389 km^2^ and more than 1.23 million inhabitants. This city is identified as one of the major social-economic, cultural, religious, educational regions of Vietnam, as well as the Mekong Delta. However, floods due to seasonal high river discharges upstream, high tides and high rainfall intensity are a significant issue that Can Tho is facing [15,16]. In addition, water pollution from untreated wastewater and illegal garbage discharge is another problem of this city [17]. During flooding time, polluted water causes some health-related issues to local people, for example diarrhea. As a 2015 local social-economic survey indicated, diarrhea and related gastrointestinal diseases are the third common diseases that people get infected with [18]. It will be even worse in the future e.g., due to climate change [8,10]. Moreover, there is evidence that fast population growth and urbanization process have led to increased flooding and decreasing water quality in Can Tho City [15,16]. 

Several studies have assessed the public health risks due to waterborne pathogen exposure cases from flooding events. Epidemiology approaches have been applied to analyze the health outcomes of flood risks. For instance, the epidemiological evidence of diarrhea was found out due to flood events at a global scale [19]. Some others suggest using the Disability Adjusted Life Years (DALYs) method to estimate the total disease burdens related to waterborne pathogens, for example: exposure to contaminated drinking water in poor urban areas in Uganda [20] or Ghana [9]. DALYs are used quite commonly by the World Health Organization (WHO) to calculate the burden of diseases regarding to hazard exposures, especially microbial hazards. Fewtrell et al. [7] determined DALYs due to flooding events for some case studies in the UK, which included deaths, serious injuries, other physical symptoms and mental health symptoms [7]. Another approache like Haas et al.’ [21] apply quantitative microbial risk assessment method (QMRA) to estimate the health risk associated to microorganism exposure. QMRA helps to predict the potential risk of diseases at a low level of concentrations, which are not easy to identify by epidemiological studies. For example, during flooding related to sewage overflows and heavy rainfall in The Hague (The Netherlands), people may get infected by *Cryptosporidium* and *Giardia* with an annual infection risk of around 5 × 10^−6^ and 3 × 10^−2^, respectively [22]. In urban areas, children playing in floodwater and swimming/rowing in river can get the highest infection probability related to gastrointestinal and Legionnaires’ diseases during flooding time [23]. Besides, infection risks are also due to flooding related to combined river water and sewage overflow [7]. The origins of floodwater, for example: fluvial, pluvial or a combination of them, affect the pathogen concentrations which is the main cause of waterborne diseases. The quantitative risk assessment, therefore, needs more considerations in order to understand the relationship between polluted floodwater and health risk.

This paper aims at providing an analysis of health risk related for a typical urban flood event in the center of Can Tho City. The analysis is a successor of a recent report on an extensive monitoring campaign of water quality dynamics during a flood event that occurred on 3 October 2013 [24]. In this study, the pathogen data of that campaign were used. 

## 2. Materials and Methods

### 2.1. Study Site

Ninh Kieu is the most urbanized and centralized district of Can Tho City. The area is a typical urban flooding site in the Mekong Delta [15]. Both pluvial and fluvial flows often occur in the city. The pluvial floods occur because the drainage network has been degraded and its capacity to deal with heavy rainfall events is limited, although in recent years, the network has been upgraded to deal with urban pluvial flooding issues [25]. In addition, the areas also suffer from fluvial floods, especially during high tide. The high tides cause inundations either because they are going through poor drainage systems where the check-valves operate improperly or because of overtopping of the river band. Both types of flooding are increasingly happening in the area. Floods happen at different places in the area. It can be either near to rivers (if the dyke is low) or inside the city through drainage/sewage networks. In this study, a monitoring campaign for a flooding event on 7 October 2013 was implemented [24]. It was one of the highest tidal−induced floods in the city in the past fifty years. 

### 2.2. Flooded Water Sampling and Analysis

**Figure 1:**
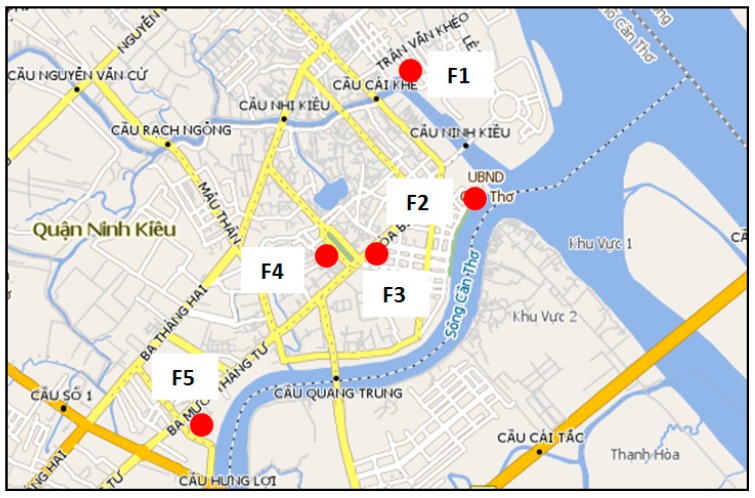
Locations of flooded sampling sites in Can Tho City.

Water samples were taken at different locations during the flood event as shown in Figure 1. The characteristics of the these five sites were different from each other. F1, F3 and F4 were more affected by overloading from drainage/sewage systems while F2 and F5 were affected because of water overtopping from the rivers. During the flood event 3–5 samples were taken at each site.

Microbiological parameters such as *E. coli*, total coliforms and *Salmonella,* among other were analyzed using the Most Probable Number (MPN) method (http://www.who.int/water_sanitation_health/resourcesquality/wqmchap10.pdf). Other water quality parameters like pH, COD, BOD_5_, nitrate (NO_3_^−^-N), ammonium (NH_4_^+^-N), phosphate (PO_4_^3−^-P), total P, total N and total suspended solids as well as hydraulic parameter such as water levels were also considered. However, as in this paper we mainly consider pathogens related to health risk issues, those parameters are omitted here and interested readers may find them in Nguyen et al. [24].

### 2.3. Quantitative Microbial Risk Assessment (QMRA)

The Quantitative Microbial Risk Assessment (QMRA) can be used to evaluate urban flood health risk, among other methods such as epidemiological population studies and comparison of the floodwater quality with the EU water standard [23]. QMRA is a technique that has been developed for calculating the burden of disease from a particular pathogen [21]. The calculation procedure consists of four successive steps: (1) hazard identification; (2) exposure assessment; (3) dose-response relations; (4) risk characterization [21].

#### 2.3.1. Hazard Identification

Identifying possible hazards in QMRA is to select possible factors that affect human health. In this study the selected pathogens were: *E. coli*, *Salmonella*, *Campylobacter*, *Cryptosporidium* and *Rotavirus*. While *E. coli* and *Salmonella* were measured in the samples, the other pathogen concentrations were calculated based on other studies. e.g., the ratio between *E. coli* and *Campylobacter*, *Cryptosporidium* is reported as 10^5^; and between *E. coli* and *Rotavirus* as 10 [9,26].

The data on pathogens in floodwater was used to identify the hazard. Because of relatively large variation in pathogen concentrations and the non-linearity of QMRA models, it is preferable to use average values with a probability distribution as inputs, rather than crisp values. For this, we had to define probability distributions of these parameters. Based on observed data of *E. coli* and *Salmonella*, a fitting function (distribution) was obtained by input of all measurement data of *E. coli* and *Salmonella* in the Easyfit software (www.mathwave.com/help/easyfit/index.html). After the distribution was obtained, a Monte-Carlo simulation of 10,000 iterations was applied, using the RiskAMP add-in for Excel software (Microsoft, Redmond, WA, USA), to generate the variations of pathogen concentrations.

#### 2.3.2. Exposure Assessment

The purpose of the exposure assessment was to determine the amount, or number, of organisms that correspond to a single exposure (termed the dose), or the total amount or number of organisms that constitute a set of exposures. We are interested in both the expected dose and the distribution of doses [27]. The exposure assessment includes identifying the ingested volume and expected dose. In this study we considered two cases: (1) adults on the street and (2) children playing with floodwater. According to ten Veldhuis [9], the mean ingested volume for a flooding event is 10 mL for adults and 30 mL for children. The dose is calculated using the following formula:

(1) $$\mu =\frac{c\times v}{d\times 1000}$$

where *c* is the concentration of a pathogen in water (number of pathogens/L), from “Hazard identification”, *v* is the ingested volume (ingested volume for adults is 10 mL and for children it is 30 mL for a flooding event) and d is the dilution factor (1 in this case as we did not dilute the floodwater). Given the concentration distribution *c* obtained after the Monte−Carlo iteration, we can also determine the dose distribution. 

#### 2.3.3. Dose-Response Relations and Risk Identification 

The dose−response relation was used to calculate the infection probability. We used the β-Poisson dose−response model for *E. coli*, *Salmonella*, *Campylobacter* and *Rotavirus* and an exponential model for *Cryptosporidium* [21] to calculate the probability of infection (P_inf_) for one event as follows. 

The β-Poisson dose—response model is:

(2) $$P_{\inf}\;=\;1\;-\;\left(\;1\;+\;\frac{\mu }{N_{50}}(2^{\frac{1}{\alpha }}\;-\;1\right){)}^{-\alpha }$$

where: μ is the dose of the pathogen (MPN), α is a parameter that characterizes the dose−response function relationship and N_50_ is the median infection dose. The value of the dose−response parameters (α and N_50_) for each pathogen are shown in Table 1.

The exponential function is:

(3) $$P_{\inf}=1-e^{-r\mu }$$

where μ is the dose of the pathogen (MPN), r is a parameter that characterizes the dose-response function relationship, and equal to 4.005 × 10^−3^.

From Equations (2) and (3) we can calculate the *Average infection probability* (P_inf_) using average concentrations of the measured and calculated data. In addition one can derive the *Infection probability distribution* using the estimated Pathogen concentration probability with the help of a Monte Carlo simulation (i.e., 10,000 iterations). 

## 3. Results and Discussion

### 3.1. Quantitative Microbial Risk Assessment

#### 3.1.1. Hazard Identification

A total of 20 water samples at five flooded sites were analyzed. From these 20 results, the measured *E. coli* and *Salmonella* distribution was fitted with a geometric function (with *p* value of 0.000055 and 0.00075 for *E. coli* and *Salmonella*, respectively). A summary of pathogen variations from measured and simulated data is shown in Table 2. *E. coli* varies from 0.0016 to 0.0007 MPN/100 mL while *Salmonella* ranges from 0.014 to 0.0091 MPN/100 mL. The differences between the measured and simulated data were not large. The mean differences were about 1 percent while the median was about 20 percent for both *E. coli* and *Salmonella*.

#### 3.1.2. Exposure Assessment

According to ten Veldhuis [9], the mean ingested volume for adults is 10 mL and children is 30 mL for a flooding event. The mean dose was calculated using Equation (1), with 10,000 iterations, and results are shown in Table 3. We can see that the average (and standard deviation—SD) dose of *E. coli* for adults as well as children is about ten times higher than *Salmonella* and about 1 × 10^3^–5 × 10^3^ MPN while the dose of *E. coli* is tenth power five, six times to *Rotaviruses*, *Campylobacter*, *Cryptosporidium*. 

#### 3.1.3. Dose-Response Relations and Risk Identification

The infection probability distributions caused by *E. coli, Salmonella, Campylobacter, Rotavirus* and *Cryptosporidium* for adults and children were estimated by running 10,000 Monte Carlo simulations. The distribution of *E. coli, Salmonella* are shown in Figure 1 while a summary of all parameters is shown in Table 4. 

**Figure 2:**
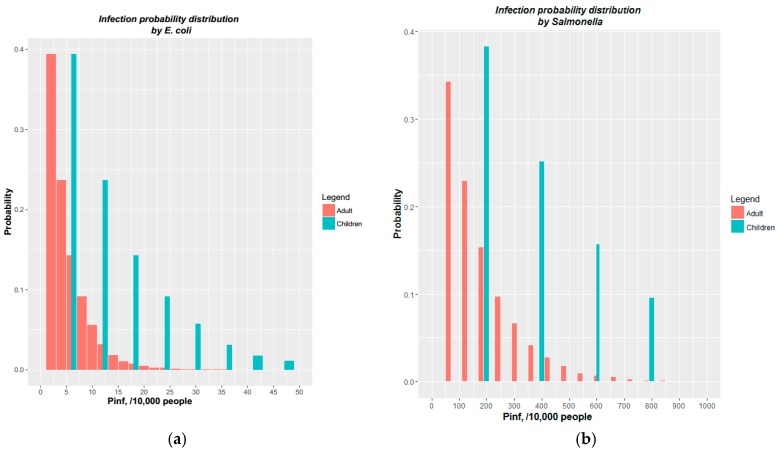
Infection probability distribution of (**a**) *E. coli* and (**b**) *Salmonella.*

It can be seen in Figure 2 that the infection probability (P_inf_) that children will get an infection is 2–3 times higher than for adults. This is because children ingest higher volumes of floodwater. For example, at the probability from 20% to 40% there are about five adults but about 10 children infected by *E. coli* and about 100 adults and 200 children infected by *Salmonella* (when over 10,000 people come into contact with the floodwater).

Though the dose of *E. coli* people receive is much higher than for other pathogens, the infection probability is lower. The mean infection probability per flood event is 4 and 12 for adults and children, respectively, with *E. coli* but it is about 137 and 374 with *Salmonella*. The infection probabilities between *E. coli* and *Campylobacter* were similar, as well as between *Salmonella* and *Rotaviruses*. However, the chance of infection of *Cryptosporidium* was low. Given 10,000 people contact to water, the maximum infected people are about 1 adult and 2 children. 

## 4. Discussion

In Vietnam, there is no standard to assess health risk when people come into contact with water. We will consider some other regulations from the Ministry of Health (MoH), Ministry of Natural Resources and Environment (MoNRE) as well as from the European Union (EU) about the water quality for different water use purposes and different ways that people may come into contact with water, as shown in Table 5. Given the range from 1.6 × 10^4^ to 7.0 × 10^4^ MPN/100 mL and from 9.1 × 10^4^ to 2.2 × 10^6^ MPN/100 mL of *E. coli* and coliform found in floodwaters, the water quality was much worse than existing water standards. It can be implied that possibly there could be serious consequences to human health. Results from this study can be considered as a first reference for Vietnamese ministries to develop such kind of flood related health risk assessment. 

The exposure of local people varied strongly during the flooding period. As observed from an interview conducted in a previous study at 34 households located near flooded areas (Figure S1 in the Supplementary Materials) [30], two thirds of the interviewees ingested floodwater while traveling on flooded streets and/or cleaning up flooded houses(Figure S2a in the Supplementary Materials). There were even some other cases where children were playing in floodwater (Figure S2b in the Supplementary Materials). Thus, cases of exposure were common in the Ninh Kieu district and a high potential for infections was found during the flood event.

It has been observed that diarrhea outbreaks increased during flooding months in Ninh Kieu district. According to Can Tho’s joint water supply—sewer company, flood events often occur from May to July and from September to November. In addition, the Can Tho Preventive Medical Center reported that diarrhea cases in Ninh Kieu district were higher from July to October (Figure S3 in the Supplementary Materials). A similar pattern was also shown in another study in the Mekong Delta, which indicated that there was a significant association between diarrhea and cumulative rainfall [31]. Furthermore, the increased health risk during flooding time in Ninh Kieu district may impact the social-economic conditions. As determined by a socio-economic survey of more than 2000 households in Can Tho City determined [32], in Ninh Kieu district only about 71% of the households have a sewer connection, and the remaining households discharge directly to the environment, e.g., a river, lake, or canal, that could be the main sources of microbial pollutants. Around 50% of households indicated that the surrounding environment has been polluted, which was related to inundation and insufficient wastewater and solid waste collection. Therefore, local people face high health risks during flooding time.

In this study, the risk characterization was assessed for a single event. This can be quantified in more detail, e.g., how many people may be infected per year or how this will change in the future. Information of population exposed to the event as well as frequency of flood events are needed. There are some studies on e.g., the exposure pathway; flooding frequencies; exposed population, that can provide more information to answer these questions [23]. In addition, epidemiological data from local health services should be exploited to assess flood-related illness information (e.g., in [33]). 

Mathematical frameworks such as QMRA are being used to estimate the infection risk from exposure to flooding in urban areas. In spite of the reliability and accuracy of this method, the infection probability of this method could be an underestimate or an overestimate given the wide range of pathogen concentrations. In most of the cases the pathogen concentration of floodwater is estimated by applying a dilution factor to the pathogen concentration at the source or sources of flooding, (e.g., in [22]). As the pathogen concentration used in such method does not consider the flood dynamics, the infection probability should be applied with care. In addition, there is an issue of parameter uncertainty. For example, the ingested volume could be a-case-specific parameter, though it was close to those reported in Fewtrell and Smith [34]. However, it should be noticed that the flood duration and transportation culture in Can Tho could be different from other places. For example, while walking and cycling are common in The Netherlands, travelling by motorbike is more popular in Vietnam. Thus, this input is still subject to uncertainty. It should be further investigated either by using distributed functions, (e.g., in [23]) or by social surveying [35]. 

“Living with floods” was a very common slogan in the Mekong Delta. It could be because people were not aware about the health risk problem or it was not really a problem in the past. The surface water has increasingly been polluted by the development of industry and urbanization. This becomes even worse if floods occur. The findings from this study may be a reference for raising awareness on health risk issues during flood events in Can Tho City as well as other similar cities in the countries of the global South.

## 5. Conclusions

In this paper, an initial assessment is presented on flood related health risk issues in Can Tho City, based on an extensive monitoring campaign. Floodwater samples were taken before, during and after flooding events at typical flooded locations in Can Tho city. By implementing the Quantitative Microbiological Risk Assessment and assuming 10,000 people come into contact with the floodwater, we found *Salmonella* is the pathogen that would cause the highest number of infections, which is about 137 and 374 in adults and children, respectively. The next most important pathogens are *Rotaviruses* (103 and 284); *E. coli* (4 and 12) and *Campylobacter* (3 and 10) per single event. *Cryptosporidium* caused the lowest infection risk with a maximum of 2 children. These values are very alarming, especially to local people, regarding health risk issues. Health risk assessment, thus, should be on the agenda of urban flood management policy. This study is the one of the first initiatives on this flood health risk in Vietnam. There should be more focus on it in the future. Besides the topic of uncertainty (e.g., the ingested volume), other aspects should also be considered, for example, pluvial flood events, advanced in-situ flood monitoring and health risk warning systems.

## Figures and Tables

**Table 1:** μ and N_50_ of *E. coli*, *Salmonella*, *Campylobacter* and *Rotavirus* [21].

Pathogens	μ	N_50_
*E. coli*	0.1952	3.01× 10^7^
*Salmonella*	0.3126	2.36 × 10^4^
*Campylobacter*	0.1450	896
*Rotavirus*	0.2531	6.17

**Table 2:** Differences between simulated (10,000 times) and observed (20 samples) *E. coli* and *Salmonella* concentration.

		*n*	Mean	SD	Median
*E. coli* (measured)	MPN/100 mL	20	1.84 × 10^4^	2.09 × 10^4^	1.00 × 10^4^
*E. coli* (simulated)	MPN/100 mL	10,000	1.83 × 10^4^	1.83 × 10^4^	1.26 × 10^4^
**Difference (%)**			0.37	14.31	20.52
*Salmonella* (measured)	MPN/100 mL	20	1.33 × 10^3^	2.03 × 10^3^	7.00 × 10^2^
*Salmonella* (simulated)	MPN/100 mL	10,000	1.34 × 10^3^	1.35 × 10^3^	9.23 × 10^2^
**Difference (%)**			1.18	50.44	24.16

**Table 3:** Average dose of pathogens per event (MPN).

	Mean		SD		Max		95 Percentile	
	Adu.	Chi.	Adu.	Chi.	Adu.	Chi.	Adu.	Chi.
*E. coli*	1.8 × 10^3^	5.5 × 10^3^	1.8 × 10^3^	5.5 × 10^3^	2.2 × 10^4^	6.5 × 10^4^	5.5 × 10^3^	1.7 × 10^4^
*Salmonella*	1.3 × 10^2^	4.0 × 10^2^	1.3 × 10^2^	3.9 × 10^2^	1.3 × 10^3^	4.0 × 10^3^	3.9 × 10^2^	1.2 × 10^3^
*Rotaviruses*	1.8 × 10^−2^	5.5 × 10^−2^	1.8 × 10^−2^	5.5 × 10^−2^	2.2 × 10^−1^	6.5 × 10^−1^	5.5 × 10^−2^	1.7 × 10^−1^
*Campylobacter*	1.8 × 10^−2^	5.5 × 10^−2^	1.8 × 10^−2^	5.5 × 10^−2^	2.2 × 10^−1^	6.5 × 10^−1^	5.5 × 10^−2^	1.7 × 10^−1^
*Cryptosporidium*	1.8 × 10^−3^	5.5 × 10^−3^	1.8 × 10^−3^	5.5 × 10^−3^	2.2 × 10^−2^	6.5 × 10^−2^	5.5 × 10^−3^	1.7 × 10^−2^

Note: Adu.: Adults; and Chi.: Children; the mean, SD, Max 95 percentile was calculated based on results of 10,000 iterations.

**Table 4:** Summary of infection probability of *Salmonella*, *E. coli*, *Rotavirus*, *Campylobacter* and *Cryptosporidium* (over 10,000 people in contact with the floodwater).

	Mean		SD		Max		95 Percentile	
	Adu.	Chi.	Adu.	Chi.	Adu.	Chi.	Adu.	Chi.
*E. coli*	4	12	4	12	36	105	12	35
*Salmonella*	137	374	131	329	1005	2196	402	1040
*Rotaviruses*	103	284	98	252	793	1769	299	786
*Campylobacter*	3	10	3	10	31	91	10	31
*Cryptosporidium*	0.08	0.25	0.08	0.25	0.74	2.22	0.24	0.73

Note: Adu.: Adults; and Chi.: Children; the mean, SD, Max 95 percentile was calculated based on results of 10,000 iterations.

**Table 5:** Water quality standard for different purposes (MPN/100 mL).

	QCVN 01/2009/BYT	QCVN 08/2008/BTNMT	QCVN 10/2008/BTNMT	EU Directive 2006/7/EC
*Coliform*	50–150	2500–5000	1000	-
*E. coli*	0–20	20–50	-	200–500 (*)

QCVN 01/2009/BYT: Domestic water for e.g., washing, bathing (but not for drinking); QCVN 08/2008/BTNMT: Inland surface water quality for domestic use, A1–A2; QCVN 10/2008/BTNMT: Coastal water quality for bathing; EU Directive 2006/7/EC: Bathing water quality [28]. (*): Converted from CFU/100 mL to MPN/100 mL by ln(MPN) = lna + b × ln(CFU), with “lna” equals to 1.27; −0.51; −1.23; 2.09 and “b” equal to 0.8; 1.04; 1.36; 0.36 for spring, summer, autumn and winter, respectively [29].

## Supplementary Materials

The following are available online at www.mdpi.com/1660-4601/14/12/1485/s1, Figure S1: Locations of the 34 households interviewed, Figure S2: Ingested flood water while (a) walking in a flooded street; (b) playing on a flooded street, Figure S3: Number of diarrhea cases during 2014, 2015 (Source: Report of Can Tho Preventive Health Care).
