# Supplementary material for: Microbial Risk Assessment of Tidal−Induced Urban Flooding in Can Tho City (Mekong Delta, Vietnam)
Source: Int J Environ Res Public Health. 2017 Nov 30;14(12):1485. doi: 10.3390/ijerph14121485 (PMC5750903; doi:10.3390/ijerph14121485)
Supplement: Supplementary file 1 [file ijerph-14-01485-s001.pdf]

Supplementary Information

Microbial Risk Assessment of Tidal−Induced Urban Flooding in Can Tho City (Mekong Delta, Vietnam)

Hong Quan Nguyen [1,*], Thi Thao Nguyen Huynh [1,2], Assela Pathirana [2] and Peter Van der Steen [2]

[1] Center of Water Management and Climate Change (WACC), Viet Nam National University—Ho Chi Minh City (VNU—HCM), Ho Chi Minh City 12345, Vietnam; n.huynh@un-ihe.org

[2] IHE Delft Institute for Water Education, Delft 2611 AX, The Netherlands; a.pathirana@un-ihe.org (A.P.); p.vandersteen@un-ihe.org (P.V.d.S.)

* Correspondence: hongquanmt@yahoo.com; Tel.: +84-908-275-996

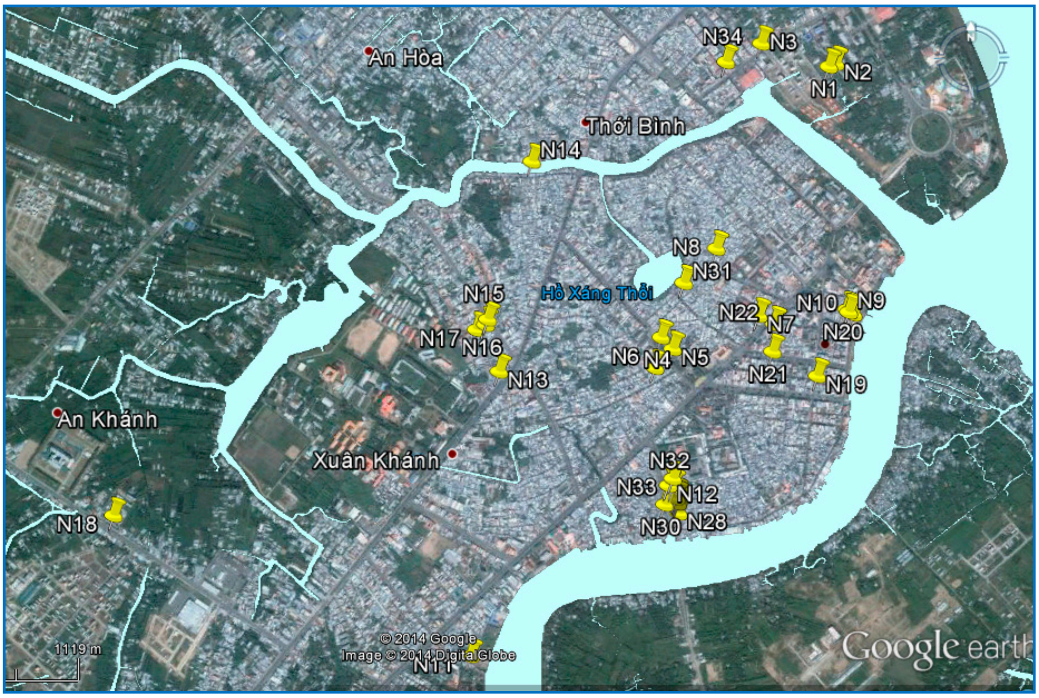

Figure S1: Locations of the 34 households interviewed

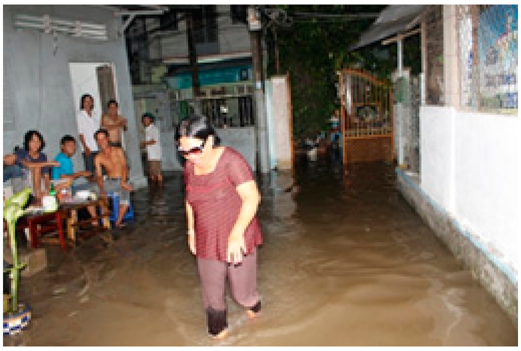

(a)

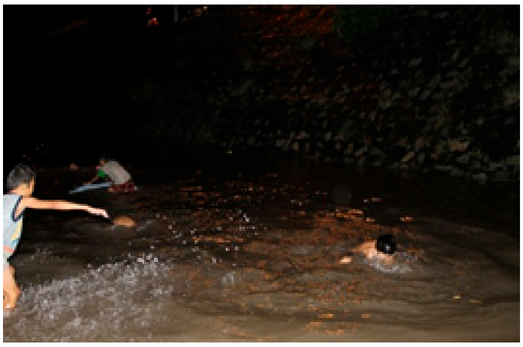

(b)

Figure S2: Ingested flood water while (a) walking in a flooded street; (b) playing on a flooded street.

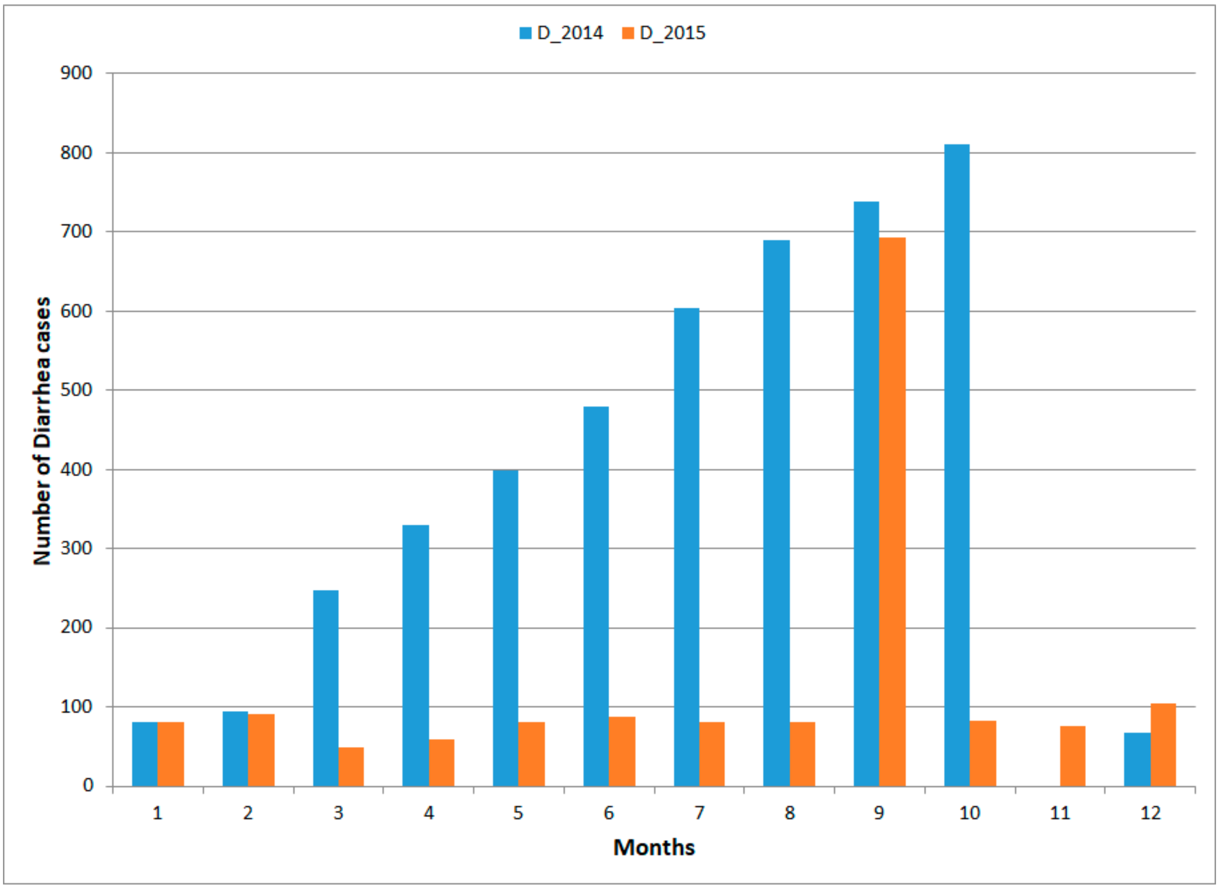

Figure S3: Number of diarrhea cases during 2014, 2015 (Source: Report of Can Tho Preventive Health Care)
